# Time dependent analysis of rat microglial surface markers in traumatic brain injury reveals dynamics of distinct cell subpopulations

**DOI:** 10.1038/s41598-022-10419-1

**Published:** 2022-04-15

**Authors:** Assaf Gottlieb, Naama Toledano-Furman, Karthik S. Prabhakara, Akshita Kumar, Henry W. Caplan, Supinder Bedi, Charles S. Cox, Scott D. Olson

**Affiliations:** 1 Center for Precision Health, School of Biomedical Informatics, University of Texas Health Science Center, Houston, TX 77030 USA; 2 Department of Pediatric Surgery, McGovern School of Medicine, University of Texas Health Science Center at Houston, 6431 Fannin St., Houston, TX 77030 USA

**Keywords:** Brain injuries, Computational models

## Abstract

Traumatic brain injury (TBI) results in a cascade of cellular responses, which produce neuroinflammation, partly due to the activation of microglia. Accurate identification of microglial populations is key to understanding therapeutic approaches that modify microglial responses to TBI and improve long-term outcome measures. Notably, previous studies often utilized an outdated convention to describe microglial phenotypes. We conducted a temporal analysis of the response to controlled cortical impact (CCI) in rat microglia between ipsilateral and contralateral hemispheres across seven time points, identified microglia through expression of activation markers including CD45, CD11b/c, and p2y12 receptor and evaluated their activation state using additional markers of CD32, CD86, RT1B, CD200R, and CD163. We identified unique sub-populations of microglial cells that express individual or combination of activation markers across time points. We further portrayed how the size of these sub-populations changes through time, corresponding to stages in TBI response. We described longitudinal changes in microglial population after CCI in two different locations using activation markers, showing clear separation into cellular sub-populations that feature different temporal patterns of markers after injury. These changes may aid in understanding the symptomatic progression following TBI and help define microglial subpopulations beyond the outdated M1/M2 paradigm.

## Introduction

Traumatic brain injury (TBI) is responsible for almost 30% of all injury-related deaths in the United States^1^. The CDC reports that 1.7 million people a year receive treatment for brain trauma^2^, and more than 5 million men, women, and children live with TBI-related disabilities in the United States. TBI can occur during many different activities at a broad range of severity levels contributing to complex cognitive and behavioral effects. Long-term, there is an additional increased risk for acquired neurodegenerative diseases including Alzheimer’s and Parkinson’s disease^3–6^. Cellular therapy has been promising in several pre-clinical studies, reducing the damage associated with neuroinflammation after TBI^7–9^. Because the injury is dynamic and has a temporal component, understanding the spatiotemporal response to TBI is vital for identifying therapeutic targets for TBI^10^ and the precise delivery of therapies^11^.

**Figure 1:**
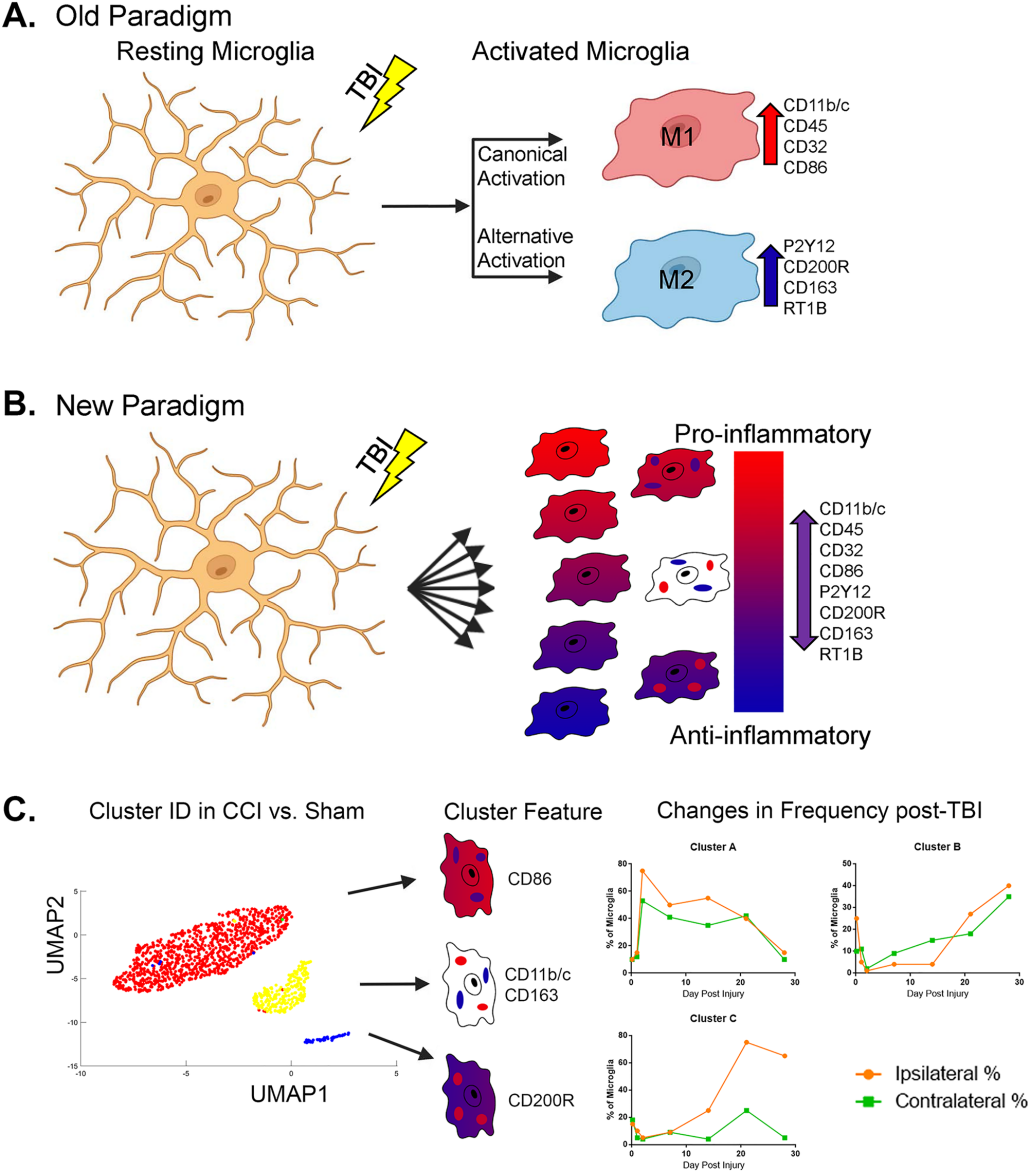
Microglial activation and process illustration. (**A**) According to the “Old Paradigm”, resting microglia are stimulated by a stimulus, such as a TBI, and proceed to become “Activated” along the “Canonical” or “Alternative” activation pathways to become either “M1” pro-inflammatory microglia with a specific surface marker profile, or “M2” anti-inflammatory microglia with a different surface marker phenotype. (**B**) In the “New Paradigm”, microglia are activated across a spectrum of pro- and anti-inflammatory activities and capable of expressing many combinations of surface markers according to cellular functions and not tied to a single pro- or anti-inflammatory phenotype. (**C**) We used cluster identification algorithms to find unique populations present in controlled cortical impact injured rats compared to sham-injured controls. These clusters were then characterized on the basis of their unique surface marker features. Finally, the changes in the distribution of microglia in each subpopulation was determined over time in both ipsilateral and contralateral hemispheres (simulated data displayed).

Microglia are important for proper function in both healthy and injured brains. Normally, microglia serve to monitor tissue for signs of injury or infection and remove cellular debris^12^. Microglia rapidly change their gene expression profile and cellular behavior within minutes of injury or infection. Activated microglia have historically been categorized similar to macrophage in activation profiles (M1/M2) based upon an idealized polarization response to inflammatory cytokines in vitro towards pro- or anti-inflammatory activity^13,14^ (Fig. 1A). According to the previous conventions, the classical activation profile (M1) is a pro-inflammatory phenotype arising in response to TNF-α and IFN-γ. The M1 pathway is associated with phagocytosis, ROS release, and release of inflammatory cytokines to combat pathogens. Activated M1 microglia upregulate their cell surface markers, such as MHC-II and CD86, to function as antigen presenting cells and interact with T cells^15^. The alternative M2 pathway has a diverse phenotypic profile in microglia but is considered anti-inflammatory due to their cytokine profile and the reported ability to protect or repair the central nervous system (CNS)^16^. However, this convention does not reflect the biological diversity of microglia in vivo, where a more continuous spectrum of activities, cytokines, and cell behavior occurs in response to a dynamic inflammatory environment^17,18^ (Fig. 1B).

There is considerable interest in better understanding the heterogeneity of microglia as a way to improve our understanding of neuroinflammation and for therapeutic gain. Single cell analysis using either RNA sequencing^19^ or mass spectrometry^20^ has enabled new ways to describe a variety of known and previously unknown microglial subpopulations on the basis of their transcriptome and proteome, respectively. These powerful techniques are also expensive, unwieldy, and difficult to interpret into previous work on microglial activity as it relates to CNS trauma and inflammation^21^.

In previous work, we studied an experimental rat model of TBI using an optimized protocol developed in our lab for multi-color flow cytometry measurements^22^, and found that the expression of purinergic receptor P2y12 can be used to distinguish microglia from macrophages^23–25^. We found that a cortical contusion in the brain results in a phenotypic change in microglia across both ipsilateral and contralateral hemispheres, with each side developing their own unique phenotypic arrangement within 24 h of injury. Our work highlights the differences between the local neuroinflammatory responses in the injured microenvironment compared to diffuse and systemic inflammation across the CNS. Here, we use multicolor flow cytometry to study the spatiotemporal responses in rat microglia surface marker expression following controlled cortical impact (CCI) injury to the ipsilateral and contralateral hemispheres across seven time points ranging between three hours and 28 days. Using a combination of statistical and unsupervised learning techniques, we identified distinct cellular sub-populations based on single or combined markers. We demonstrate that these sub-populations persist though time but that their prevalence in each time point changes, reflecting development of the microglial response to CCI over time.

## Results

### Microglial populations activated after CCI

For the remaining analysis, we rely on our microglia identification technique (Methods) and focus on analysis of microglia cells in both ipsilateral and contralateral locations that show differentiated markers relative to sham, across seven time points: 3 h, 1, 2, 7, 14, 21 and 28 days following CCI. The overall number of cells with differentially expressed profiles are displayed in Table 1, encompassing a fraction of 1.6% and 1.8% of the measured cells for ipsilateral Panel A and B markers, respectively, and 2.3% of the cells for contralateral Panels A and B markers each.

**Table 1:** Description of identified cell sub-populations with their most prominent markers (mentioning markers with median > 3 standard deviations).

Panel A				Panel B			
Cluster #	Number of cells		Overexpressed markers	Cluster #	Number of cells		Overexpressed markers
	Ipsi	Contra			Ipsi	Contra	
**1**	668 (119 fold)	550 (90 fold)	P2Y12 (p < e^−108^)	**1**	644 (109 fold)	421 (92 fold)	P2Y12 (p < 2e^−43^)
**2**	258 (13%)	200 (24%)	CD11 (p < 3e^−103^)	**2**	205 (6%)	61 (13%)	CD11 (p < 9e^−34^)
**3**	143 (32%)	207 (7%)	CD45 (p < 3e^−61^)	**3**	75 (0%)	51 (8%)	CD200R (p < 4e^−32^)
**4**	130 (15%)	177 (12%)	CD32 (p < 2e^−76^)	**4**	63 (11%)	136 (6%)	CD11, CD163 (p < 8e^−25^ , p < 5e^−39^, respectively)
**5**	72 (0%)	50 (0%)	CD86, CD45 * (p < 7e^−29^, p < 9e^−8^, respectively)	**5**	341 (306%)	105 (59%)	RT1B § (p < 2e^−53^)
				**6**	^−^	164 (9%)	CD45 (p ~ 0)

Numbers of cells included in sub-populations. Percent of additional cells in the expanded groups in parenthesis.

*CD45 less pronounced in contralateral.

^§^RT1B less pronounced in ipsilateral.

Our analysis include the following three steps (Fig. 1C, Methods):

1. Identification of sub-populations of microglial cells that are statistically different from sham injury based on measured markers across the seven time points (termed “core groups”).
2. Explore time-dependent trends in sub-population sizes in the core groups.
3. Explore expansion of these sub-populations to microglial cells with similar profiles to the core groups, but lower marker activity.

### Microglial marker-based sub-populations

#### Identifying cells different from sham

Our first step was to identify microglial cells that are statistically different from sham in ipsilateral and contralateral areas, respectively, based on single and combination of markers. We provide analysis of the markers in each panel independently, although some markers (specifically P2Y12, CD11 and CD45) were present in both marker panels. We aggregated cells that expressed markers and marker combinations significantly different from sham across all time points (Table 1, “Methods”). Interestingly, the fraction of significantly different cells in the ipsilateral location were lower than the fraction of significantly different cells in the contralateral location (1.6%-1.8% vs. 2.3%).

#### Detecting sub-populations

Using the algorithm of Quantum Clustering (QC)^26^ across multiple resolutions, we identified that the most robust number of clusters occurred between 5–6 clusters (specifically, five clusters for ipsilateral Panel A and B and contralateral Panel A markers, and six clusters for contralateral Panel B markers) (Figure S1). Table 1 displays the number of cells in each cell group. We verified that these clusters are robust by visualizing them using t-SNE^27^ and UMAP^28^ dimensionality reduction methods (Figs. S2–S5).

#### Characterizing cell sub-populations

**Figure 2:**
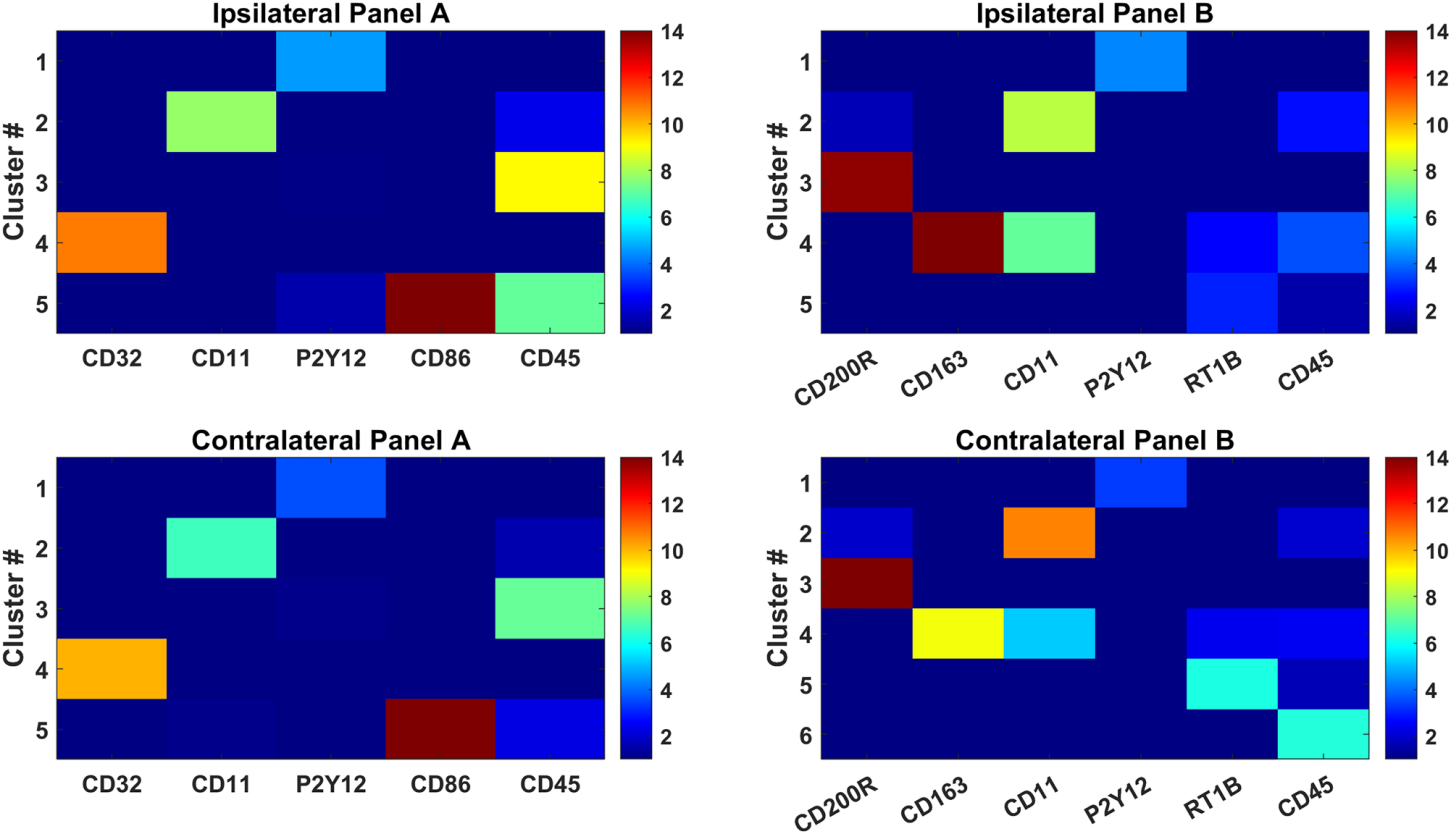
Cluster signatures over Panel A and B markers. Median expression values for ipsilateral clusters and contralateral clusters depict the most prominent markers associated with each cluster.

The identified cell clusters were characterized by single or combination of markers that differentiated them from other clusters (statistical significance using Wilcoxon ranked sum test). In the following, we describe these microglia sub-populations (clusters shown in Figs. 2 and S6-S9) in terms of their defining markers. The majority of the sub populations are defined by a single marker. These include cluster #1 displaying elevated expression levels of P2Y12 (measured in both panels, Table 1), cluster #2, which exhibits elevated levels of CD11 in both panels and cluster #3 in Panel A/cluster #6 in contralateral Panel B that display elevated CD45. Notably, ipsilateral Panel B does not exhibit a sub-population featuring only CD45 without other highly expressed markers. Two additional sub-populations that overexpress markers unique to the two panels, namely cluster #4 in Panel A displays elevated CD32 and cluster #3 shows elevated CD200R in Panel B.

Few clusters show elevated expression of two or more markers. Specifically, cluster #4 in Panel B co-expresses CD11 and CD163 and cluster #5 in Panel A overexpresses both CD45 and CD86. Notably, in the sub-population co-expressing CD86, CD45 is much more pronounced in ipsilateral than contralateral (Fig. 2). It should be noted that CD45 is co-expressed with other markers in other clusters, namely CD11 and CD163 in cluster #2 and #4 of Panel B, but CD45 expression is much less pronounced than the other markers in each of these sub-populations.

### Proportions of microglial sub-populations change through time following CCI

**Figure 3:**
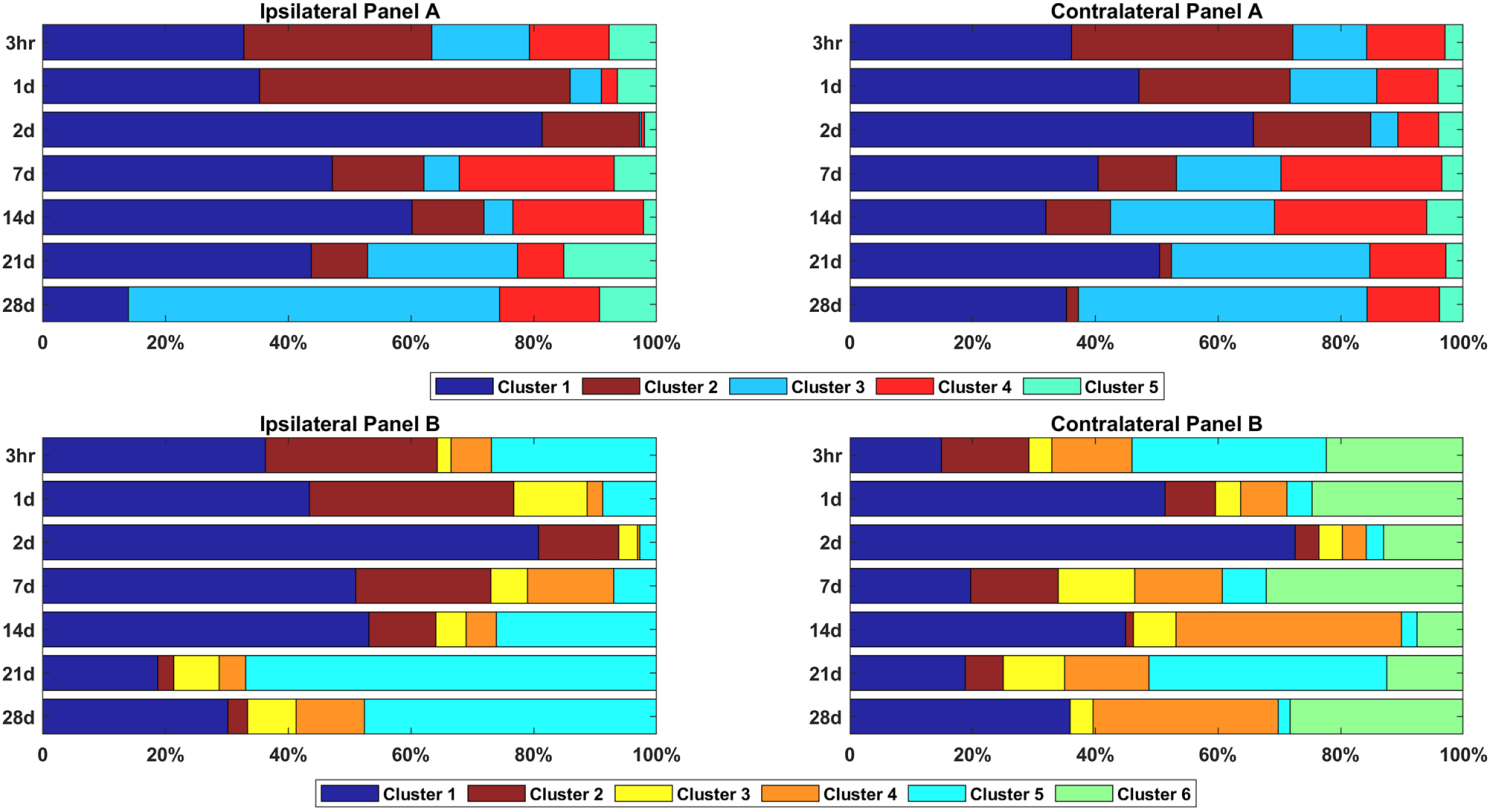
Fraction of cells in each cluster over the seven time points. The fraction of cells in each cluster out of the entire set of cells differentially expressed from sham for ipsilateral clusters and contralateral clusters. Colors denote clusters with similar marker profiles, where clusters 1 and 2 have the same profile (P2Y12 and CD11) across Panel A and Panel B.

Most identified sub-populations include cells from all time points. However, the fraction of cells belonging to each sub-populations (out of the entire repertoire of cells different from sham) varies significantly between time points (Fig. 3). In the following, we describe the most prominent changes through time in sub-populations and their expressed markers.

#### P2Y12 (cluster 1)

The fraction of cells in cluster no.1, overexpressing P2Y12, increases from 15 to 36% of the cells at three hours post CCI (in both ipsilateral and contralateral) to a maximum of 81% in ipsilateral and 66–73% contralateral on day 2. This fraction remains elevated through 21–28 days in the ipsilateral hemisphere, compared to only 7–14 days in the contralateral hemisphere. This cluster is consistent with microglial activation and expansion as a result of injury.

#### *CD11 (clusters 2 in panel A and 2* + *4 in panel B)*

A similar, but earlier pattern to cells overexpressing P2Y12, is observed with cluster no. 2, including CD11-overexpressing cells. In ipsilateral, the portion of these cells reaches 51% on day 1 and gradually reduces in subsequent time points until completely vanishing on day 28. Different from ipsilateral, in contralateral the fraction of CD11-overexpressing cells start decreasing already after 3 h, and do not peak on day 1 as in ipsilateral. Interestingly, an additional cluster in Panel B that include combination of CD11 with CD163 (cluster #4) displays different patterns, where the fraction of cells expressing both CD11 and CD163 reduces from 3 h to reach a minimum on day 2, increasing in later days to fractions larger than the 3 h baseline. This cluster phenotype is consistent with the more classical “M2” phenotype that conventionally indicates anti-inflammatory activity.

#### CD45 (clusters 3, 5 in panel A, cluster 6 in contralateral panel B)

The fraction of cells expressing CD45 in Panel A set reduces to minimum (< 1% in ipsilateral and 4% in contralateral) on day 2, followed by gradual increase until reaching between 60% (ipsilateral) to 47% (contralateral) of the cells, surpassing the fraction of cells observed at three hours. Interestingly, the proportions of cells of the CD45 cluster in ipsilateral decline faster than in contralateral (decrease observed already in day 1 vs. day 2 in contralateral) and much slower increase in fraction after day 2 in ipsilateral than in contralateral, surpassing the fraction observed at three hours only after 21 days, vs. 7 days in contralateral. This result displays earlier response than was reported in Truettner et al., where they reported that the number of CD45 high expressing cells began to decrease only after three days^29^. Moreover, the fraction of CD45 expressing cells in Panel A reaches a large fraction of the cells (60% in ipsilateral and 47% in contralateral). However, less consistent patterns are observed in a cluster of cells expressing CD45 that is unique to contralateral in the Panel B set of cells and a cluster that co-expresses CD45 and CD86 (cluster #5 in Panel A).

#### CD32 (cluster 4 in panel A)

The pattern of CD32 in ipsilateral shows an initial response of reduction in cells expressing CD32 until day 2, followed by large increase in the fraction of the cells of this sub-population on days 7 and 14, then falling back to the 3 h baseline after 21 days. The expression pattern and phenotype are consistent with a conventional “M1” pro-inflammatory phenotype for microglia.

#### RT1B (cluster 5 in panel B)

There is a large difference in the patterns of cells expressing RT1B in ipsilateral and contralateral. While in ipsilateral the fraction of cells expressing RT1B reduces to minimum at day 2 and go back up, in contralateral this pattern is not very pronounced.

#### CD200R (cluster 3 in panel B)

The fraction of cells expressing CD200R remains relatively constant across time points.

### Expansion of sub-populations

While the cells that differentiate from sham display clear sub-populations, we hypothesized that these microglial cells draw from a pool of cells that have more moderate response to CCI and do not pass significance threshold from sham. We thus sought to estimate how large this pool of additional moderate-response cells is.

We used the core groups as seeds and expanded these clusters based on proximity to the center of each sub-population (Methods). Most sub-populations gained up to 32% increase in size with potentially similar cells, with some clusters gaining none (e.g. cluster #5 in Panel A and cluster #2 in ipsilateral Panel B, Table 1), suggesting that the majority of cells are already significantly different from sham. Two notable exceptions are cluster #5 in Panel B, expressing RT1B, with anywhere between 59% (contralateral) to 306% (ipsilateral) additional cells that could be attributed to this sub-population and the clusters that express P2Y12. Expanding this cluster incorporates the majority of measured cells, reaching low expression levels, that suggests that the majority of cells do not participate in the response to CCI. We verified that clusters including these moderate marker expressing cells remain well separated (Figures S10-S13). Corresponding to the core clusters, the expanded clusters retained high fold change in their markers relative to other clusters (fold change > 51 for all markers except for the expanded cluster #1, which incorporates multiple cells with low expression of all markers).

## Discussion

In this study, we examined microglia cells across different time points to determine how a focal cortical contusion in the brain results in time-dependent changes in the markers that microglial cells express, and how they differ between ipsilateral and contralateral hemispheres**.** We use our previously established techniques to identify microglia based on morphology and specific markers (CD45^+^CD11^+^P2y12^+^ cells) and provide high-resolution insight into both ipsilateral and contralateral microglial marker expression after injury using multi-color flow cytometry-based data analysis**.** We focused here on cells that had marker profiles distinct from sham and expanded them to similar cells. We showed that only a small fraction of the microglial cells are distinct from sham, ranging between 1.8% in ipsilateral and 2.4% in contralateral. Several clustering analyses confirmed that these cells cluster into distinct sub-populations that dynamically change in size through the activation timeline of the microglia.

Microglia response to trauma is dynamic. Previous reports that relied upon the antiquated M1/M2 convention have found that microglia first respond to TBI with a transient anti-inflammatory (“M2”) phenotype lasting up to a week, followed by a sustained pro-inflammatory (“M1”) phenotype that can last for months and years^30–32^. It has become clear that microglial phenotypes and functions are not restricted to a simple pro- or anti-inflammatory activity. Here, we sought to better understand the temporal dynamics of microglial subpopulations using surface marker panels that have conventionally been associated with M1/M2 phenotypes using paradigm-agnostic methods. Since both panels overexpress some of the same markers, we built a composite expression profile to better suggest/support one archetype over another. Due to changes in the injury microenvironment, microglia and systemic macrophages should dynamically react to different stimuli to result in a unique expression profile, which can change over time or with treatments^33^. This concept has also been previously reported in other acute CNS injury models, such as spinal cord injury and ischemic brain injury^31,32^.

We found that the specific markers used to identify the cells changed following activation as a result of brain injury. In a previous study, our analysis focused on the effects of TBI on microglia at 24 h and proposed only a narrow view of the change. Our new analysis suggests that through activation, different sub-populations express different combinations of these markers but the fraction of cells from each sub-population changes in a spatiotemporal manner. This directly supports the idea that there are time dependent patterns in microglial phenotypes and activations with generally more pronounced responses in ipsilateral samples than contralateral in both pro- and anti-inflammatory markers.

An interesting and unexpected observation, we found that the fraction of significantly different cells in the ipsilateral hemisphere were lower than the fraction of significantly different cells in the contralateral hemisphere (1.6–1.8% vs. 2.3%). Our study was not designed to robustly quantify this metric due to the number of processing steps involved in tissue dissociation, cell isolation, and analysis method. Other studies have reported that the sham injury procedure can cause low level neuroinflammation particularly when the sham injury incorporates a craniotomy^34,35^. Another possibility is that the sham injury surgical procedure itself may affect the ipsilateral side with regard to inflammatory reactions compared to the contralateral side. Studies have described channels directly connecting the skull and the brain alongside the burgeoning field of glymphatics is describing new possibilities for dynamic communication between tissues on both sides of the blood–brain barrier^36,37^. These mechanisms could affect the number and distribution of unique microglial phenotypes when comparing CCI to a sham injury.

Moore et al., previously found that the expression of P2y12 increases on anti-inflammatory microglia compared to surveying microglia or microglia activated to a pro-inflammatory phenotype in vitro using human fetal and adult microglia cells^38^. This corresponds well with our observation that the fraction of cells increases in the early phase, peaking in 48 h past CCI, and gradually decreases after that, with differences between ipsilateral and contralateral hemispheres, where the return to baseline fractions is much slower on the ipsilateral side.

Antigen presenting cells express both CD86 and RT1B and help mediate microglial interactions with T lymphocytes^12^. T cell infiltration was found to be transient at 5–7 days post injury^39^, delaying the expression of these markers on microglia subsets. This corresponds well to the pattern of ipsilateral RT1B expressing cells, where a gradual increase in the fraction of these cells is observed beginning on day 7, but no clear pattern is observed on the contralateral side, suggesting a subtle effect in a more distant location from the injury. For cells co-expressing CD86 and CD45, we do see an increase in the ipsilateral fraction of cells only around day 21 and again no increase in the contralateral hemisphere.

Conventionally, the microglial CD200 receptor (CD200R) binds the neuronal ligand CD200 preventing activation^40,41^. In vitro studies have found that CD200R expression is associated with microglia activation on an anti-inflammatory subpopulation^41^. In our study, the fraction of cells expression CD200R remained relatively constant across all time points.

A classically pro-inflammatory marker, CD32, participates in inflammation regulation and phagocytosis^42^, however the specific role of CD32 expression in response to TBI is not clear. In one study, CD32 expression was shown to increase in both mRNA and protein levels 24 h after TBI in a mouse model, with peak expression at day 5^30^. This only partially agrees with our observations, where we observe decrease in the fraction of CD32 expressing cells until day 2, but indeed a peak in the number of expressing cells on days 7–14.

One of the more common anti-inflammatory markers associated with phagocytosis, CD163, has been associated with the “M2” activation pathway^43,44^. Zhang et al., previously described an accumulation of CD163^+^ cells at the TBI lesion within 48 h in a rat model^45^. Similarly, Turtzo et al., also examined CD163 expression of RNA and protein in macrophage and microglia in rats following CCI, finding that initial low levels of both RNA and protein expression gradually increased to a peak level 5 days after injury^46^. We observe that the fraction of CD163 expressing cells diminishes towards day 2 and increases back on day 7. Here, ipsilateral and contralateral hemispheres diverge. On the ipsilateral side, the CD163^+^ fraction decreases on day 14, but on the contralateral side it continues to be high throughout the time course of 28 days. Our method is capable of improving on these studies by specifically profiling microglia separate from infiltrating myeloid cells.

## Conclusions

Using clustering analysis, we identified sub-populations of microglial cells that change over time following the injury. These clusters based on the markers mean fluorescence intensity were clear and their appearance was mostly consistent with the literature. Our results show time-dependent shifts in the proportions of each cellular sub-population and support difference in effect between ipsilateral and contralateral hemispheres. Taken together, our work shows how microglia cells change following CCI and could be used in the future to develop targeted treatment for each stage of the microglial changes.

## Methods

### Animals

Male Sprague Dawley Rats (225–250 gr, Envigo Labs) were the source of CNS tissue. The usage of the animals was approved by the Animal welfare committee at University of Texas Health Science Center at Houston, Texas, protocol: AWC16-0046. Animals were handled in accordance with the standards of the American Association for the Accreditation of Laboratory Animal Care (AAALAC). Five-week old rats were housed in pairs under 12 h light/dark cycles in temperature-controlled conditions. Water and standard rodent laboratory chow were accessible ad libitum.

### Controlled cortical impact model

We utilized an experimental model of TBI as previously described^47–50^. To establish TBI model in the rats, unilateral brain injury was induced using Impact One Stereotaxic Impactor (Leica Microsystems, Buffalo Grove, IL) a controlled cortical impact (CCI) device. Rats were anesthetized with 4% isoflurane/O_2_. After mounting their head in a stereotactic frame and performing a craniotomy to expose the dura, they received a single impact to the right parietal association cortex of 1.0 mm depth of deformation with an impact velocity of 5.0 m/sec and a dwell time of 200 ms using a 6 mm diameter impactor tip to achieve moderate to severe injury. Alternatively, a sham-injury control procedure was performed by similarly exposing the skull without a craniotomy or impact. The injury procedure was complete after the incision was closed with staples. Three rats (N = 3) were used for each injury (Sham, CCI) at each time point (3 h, 1d, 2d, 7d, 14d, 21d, and 28d) for a total of 42 rats.

### Tissue dissociation and cell isolation

To apply flow cytometric methodology for cell characterization we dissociated the brain tissue into single cell suspension. Twenty-four hours after injury, rats were anesthetized with 4% isoflurane/ O_2_, and sacrificed via right atrial puncture. Brains were excised and the cerebellum was removed. Brains were divided to ipsilateral and contralateral hemispheres, for separate processing. The entire brain cell population was isolated from the brain tissue by enzymatic digestion and mechanical dissociation using Neural Tissue Dissociation Kit with GentleMACS (Miltenyi Biotec), according to manufacturer protocol with a few modifications to adjust the mouse neural tissue oriented protocol to accommodate rat brain tissue. Briefly, each brain hemisphere was placed in C tube and added with pre-warmed 4 ml of Buffer X and 100 µl of Enzyme P. After mechanical dissociation with the GentleMACS on program m_brain_01, cells were incubated on a tube rotator for 15 min at 37 °C. Following program m_brain_02, cells were added with 40 µl of Buffer Y and 20 µl of Enzyme A, and incubated for 10 min as before. For the last incubation, at the same conditions, we applied program m_brain_03. Cells were filtered using a 70 µm MACS strainer, centrifuged and washed once with HBSS (Gibco). Myelin removal was achieved by suspension of each brain hemisphere in 3 ml of 30% Percoll (GE Healthcare Life Sciences) in Hanks' balanced salt solution (HBSS), placed in a 15 ml falcon tube. Cells were centrifuged at 700 g for 10 min, with no break, myelin accumulated at the top while cells were submerged into a pellet. After myelin aspiration, cells were washed in HBSS in a total volume of 12 ml to dilute the Percoll and centrifuged at 500×*g* for 10 min.

### Microglia enrichment

The cell pellet consisting of a mixture of all brain cells was further subjected to magnetic cells sorting for microglia enrichment using CD11b/c microbeads (Miltenyi Biotec), according to manufacturer protocol. The retrieved cells of the non-enriched and enriched fractions were suspended in microglia medium (ScienCell Research Labs. Inc.), and kept overnight until processing for flow cytometric analysis the next day.

### Identification of microglia

We demonstrated our methodology for detecting changes in microglial populations of ipsilateral and contralateral brain hemispheres and previously validated their accuracy at 24 h after injury in previous publications^23–25^. To specifically identify microglia and to characterize their pro- and anti-inflammatory phenotype at each of the seven time points, CD11b/c enriched cells were surface stained. The list of antibodies is summarized in our published optimized multicolor immune-phenotyping protocol^22^ and Supplementary Table S1. Each sample of the CD11b/c enriched cells was five times diluted with staining buffer (Biolegend), washed and divided to into Panel A containing surface markers previously described as pro-inflammatory and Panel B containing surface markers previously described as anti-inflammatory. Cells were incubated for 30 min at RT in the dark. After another wash, cells were added with the secondary antibody mix and Ghost live/dead reagent and incubated for 20 min at RT in the dark. Cells were finally washed, added to counting control beads (Cyto-Cal) and run. Data was acquired with LSRII cytometer with FACSDiva acquisition software version 8.0 (BD Biociences, San Jose, CA https://www.bdbiosciences.com/en-us/products/software/instrument-software/bd-facsdiva-software#Overview). Analysis was conducted using Kaluza version 1.5a software (Beckman Coulter, Brea, CA https://www.beckman.com/flow-cytometry/software/kaluza). Fluorescence spillover compensation values were generated using VersaComp Antibody Capture Beads (Beckman Coulter, Inc.). The main population of cells was identified through Forward Scattered light (FSC) and Side Scattered light (SSC), where FSC measures the relative size of the event while SSC measures the event granularity^51^. Single live cells were identified by exclusion of doublets and gating of viable cells using live/dead dye (Ghost Dye, Tonbo Biosciences, San Diego, CA) while microglia cells were identified as cells positive for CD45, CD11b/c and P2Y12.

### Computational analysis

#### Identifying cells different from sham

To identify cells that show markedly different marker profile from sham, we used a z-test on each microglial marker in each cell relative to its corresponding sham (i.e., ipsilateral and contralateral, Panel A and B relative to their respective shams). In order to capture cells without single marker deviation from sham but rather a marker combination, we performed a principal component analysis on the combined data and sham and performed z-test within the embedded space of the principal components.

#### Identifying core-groups

We used the algorithm of Quantum Clustering (QC)^26^ for the entire set of outlier cells across all the time points to identify natural grouping. QC has one parameter, which designates a resolution parameter. We preprocessed the data by using the top five principal components of the data z-score normalized data (out of seven dimensions of Panel A and eight dimensions of Panel B) followed by whitening normalization, which normalizes the sum of dimensions to 1. QC can be applied across multiple resolutions in order to identify the most robust number of clusters, one that remain constant across the maximal range of resolutions. The maximal resolution out of the range of robust clusters was selected for defining the clusters. Verifications of the independence of the computed clusters were done using t-SNE^27^ and UMAP^28^ dimensionality reduction techniques.

#### Expanding core-groups

For expanding the core groups, we assigned cluster numbers based on the closest cluster center (average of all members of the core-groups) based on Euclidean distance. The Bioinformatics analysis was performed in Matlab v9.

### Ethics approval and consent to participate

The usage of the animals was approved by the Animal Welfare Committee at University of Texas Health Science Center at Houston, Texas. Animals were handled in accordance with the standards of the American Association for the Accreditation of Laboratory Animal Care (AAALAC). The authors confirm that they complied with the ARRIVE guidelines.

## Supplementary Information

Supplementary Information.

## Data Availability

The datasets generated and analyzed during the current study are publicly available on the Zenodo platform https://zenodo.org/record/5590207 .
