# Supplementary material for: Time dependent analysis of rat microglial surface markers in traumatic brain injury reveals dynamics of distinct cell subpopulations
Source: Sci Rep. 2022 Apr 15;12:6289. doi: 10.1038/s41598-022-10419-1 (PMC9012748; doi:10.1038/s41598-022-10419-1)
Supplement: Supplementary file 1 — Supplementary Information. [file 41598_2022_10419_MOESM1_ESM.pdf]

Time Dependent Analysis of Rat Microglial Surface Markers in Traumatic Brain Injury Reveals Dynamics of Distinct Cell Subpopulations

Assaf Gottlieb [1], Naama Toledano-Furman [2], Karthik S. Prabhakara [2], Akshita Kumar [2], Henry W. Caplan [2], Supinder Bedi [2], Charles S. Cox Jr. [2], Scott D. Olson [2]

[1] Center for Precision Health, School of Biomedical Informatics, University of Texas Health Science Center, Houston, Texas 77030.

[2] Department of Pediatric Surgery, McGovern School of Medicine, University of Texas Health Science Center at Houston, 6431 Fannin St. Houston, Texas, 77030.

Supplemental Table S1. Multicolor Flow Cytometry Microglia/Myeloid Cell Panels

Antibody	Fluorochrome	Clone	Vendor	Catalog #	Purpose
**CD45**	APC-Cy7	OX-1	BD	561586	Leukocyte common antigen.
**CD11b/c**	PE-Cy7	OX-42	BD	562222	General myeloid cell marker.
**P2Y12**	BV421	n/a	Alomone Labs	APR-020-F	Mediates microglia chemotaxis.
**CD32**	PE	D34-485	BD	562189	Associated with phagocytosis, activates inhibitory signaling
**RT1B**	Alexa Fluor 647	OX-6	BD	562223	MHC class II marker, antigen presentation
**CD163**	PerCP Cy5.5	ED2	Bio Rad	MCA342R	Associated with hemoglobin clearance Microglia (+), macrophage (+)
**Ghost**	BV510	n/a	Tonbo	13-0870-T100	Viability Dye

Supplementary Figures

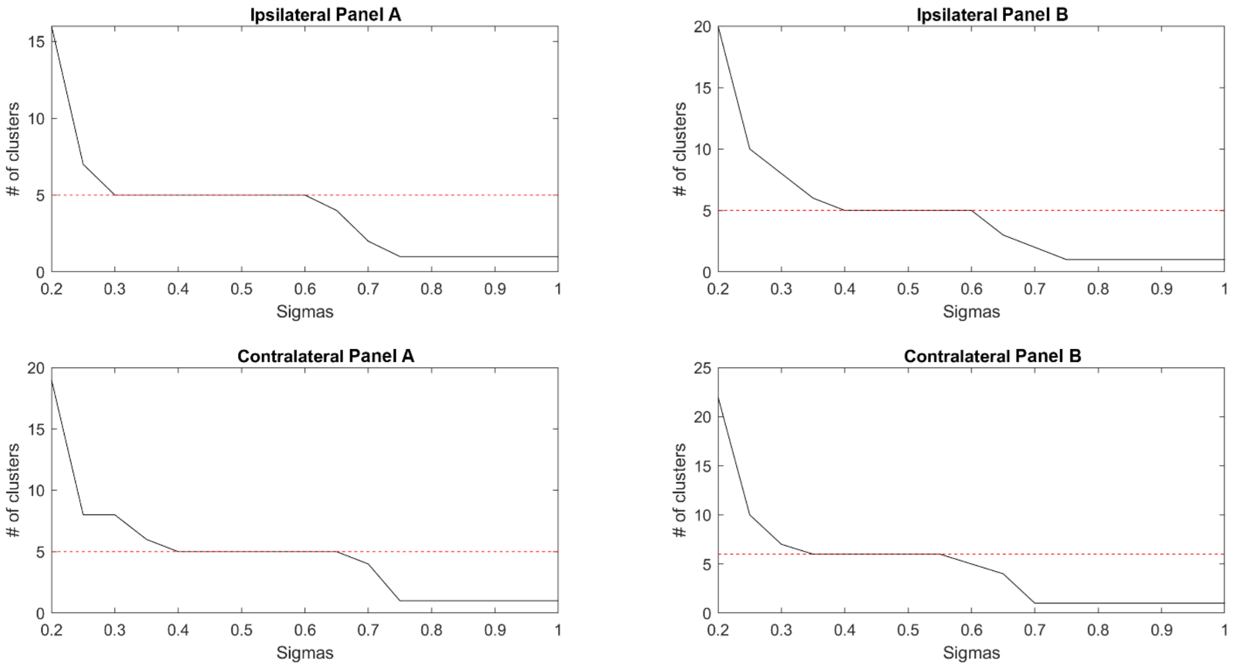

Figure S1: Cluster robustness analysis, computing the number of clusters across a large range of resolutions, where lower sigma means higher resolution. Red dashed line signifies the most robust number of clusters, while the final horizontal line is at too low resolution where all cells are on the same cluster.

A

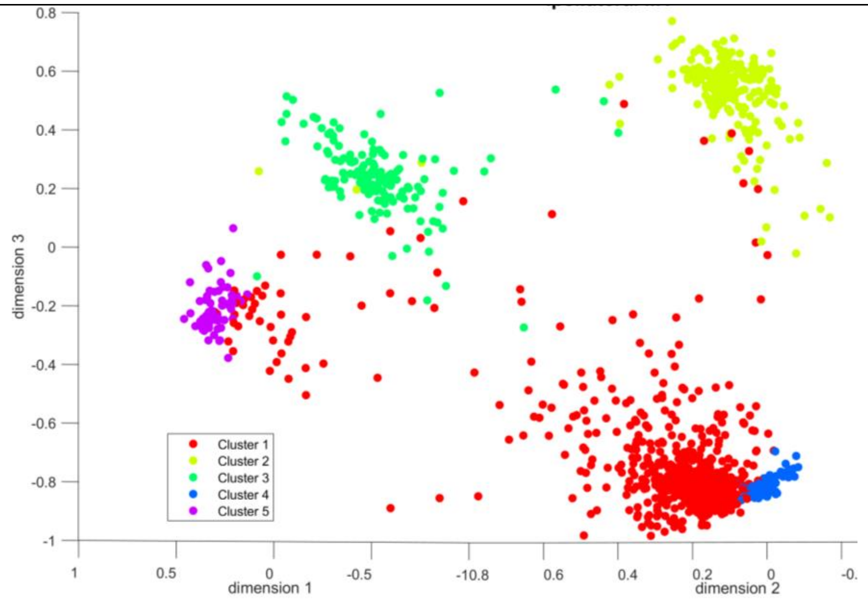

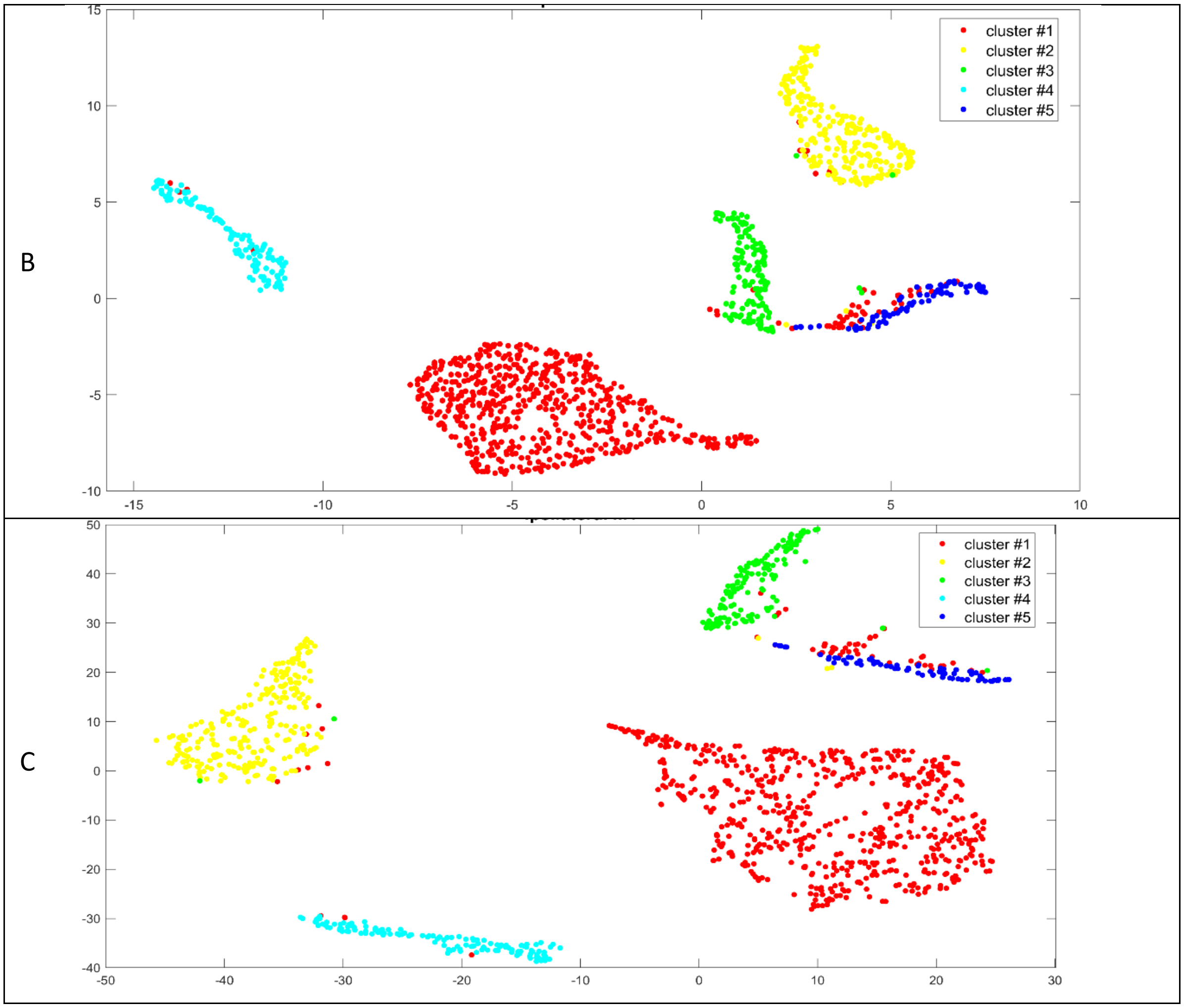

Figure S2: Robust clusters for ipsilateral Panel A using principal component analysis (A), UMAP (B) and t-SNE (C)

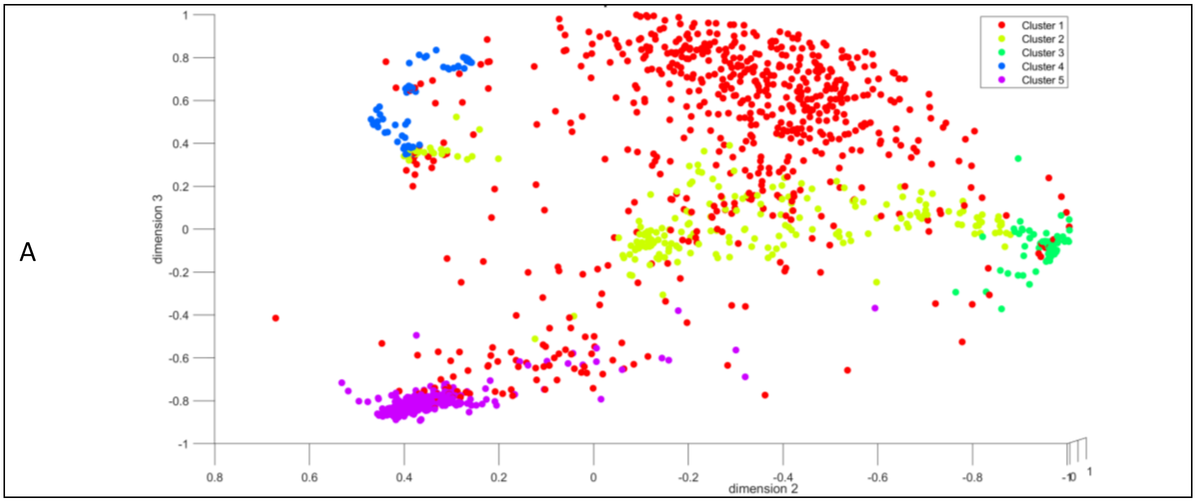

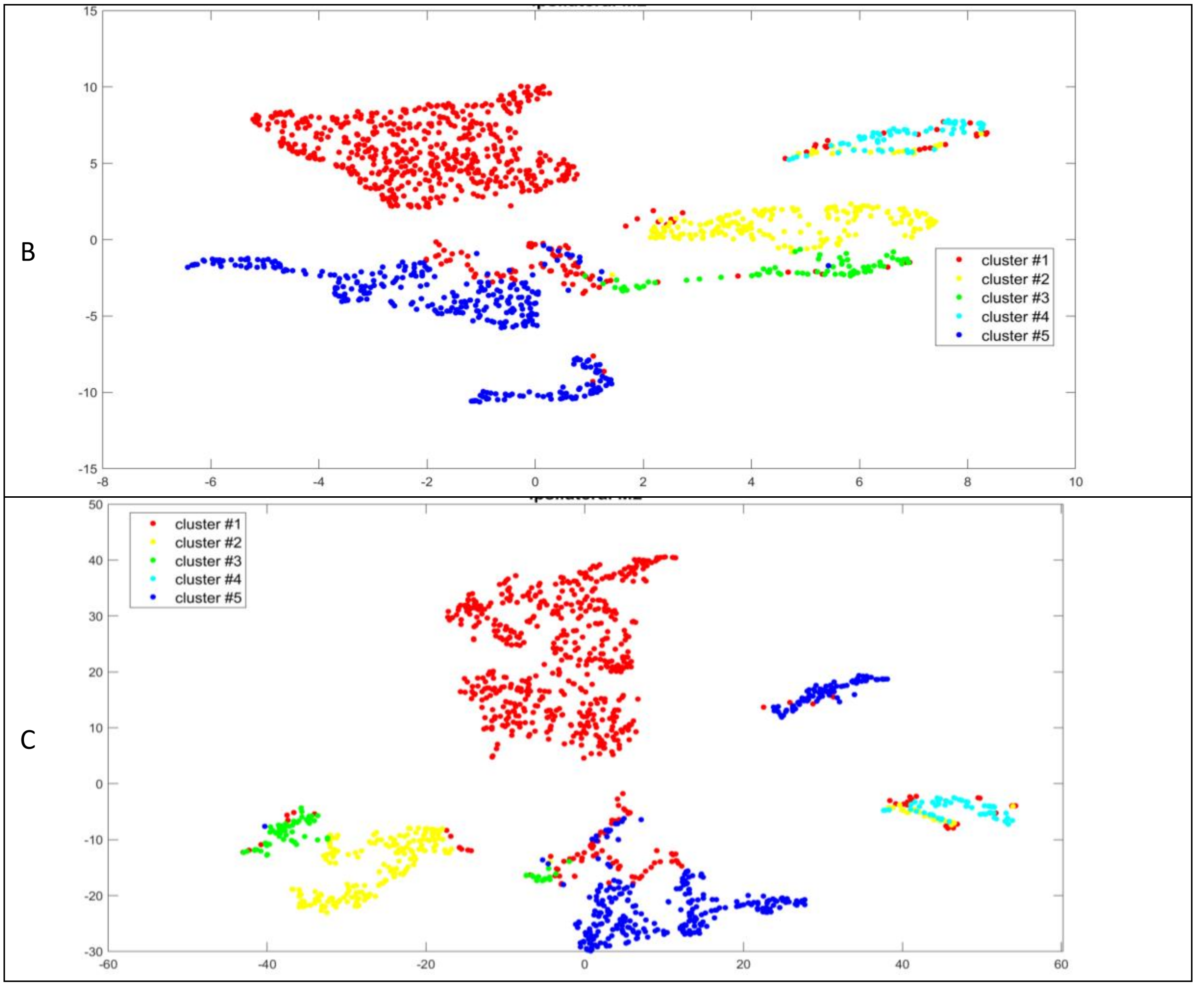

Figure S3: Robust clusters for ipsilateral Panel B using principal component analysis (A), UMAP (B) and t-SNE (C)

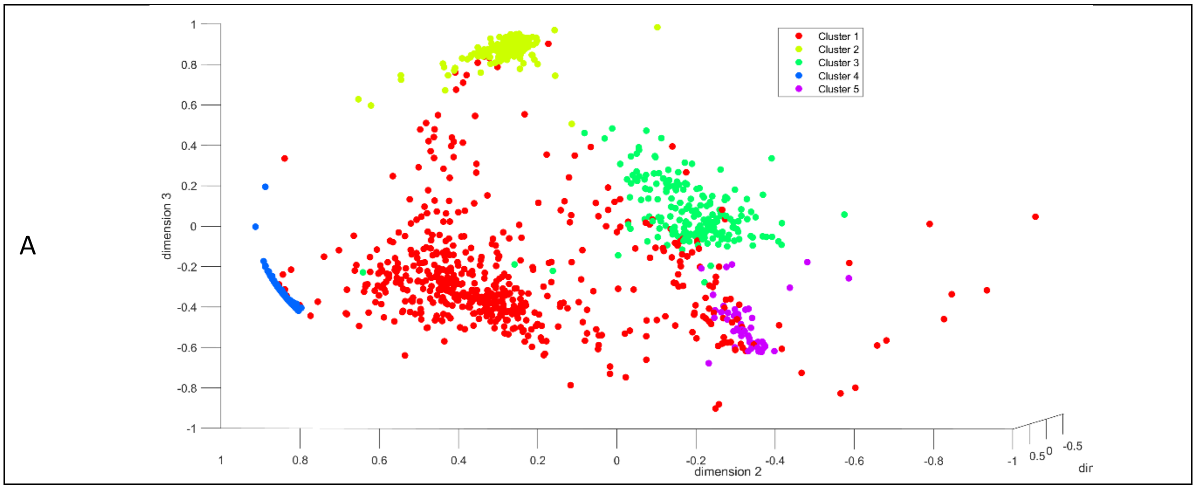

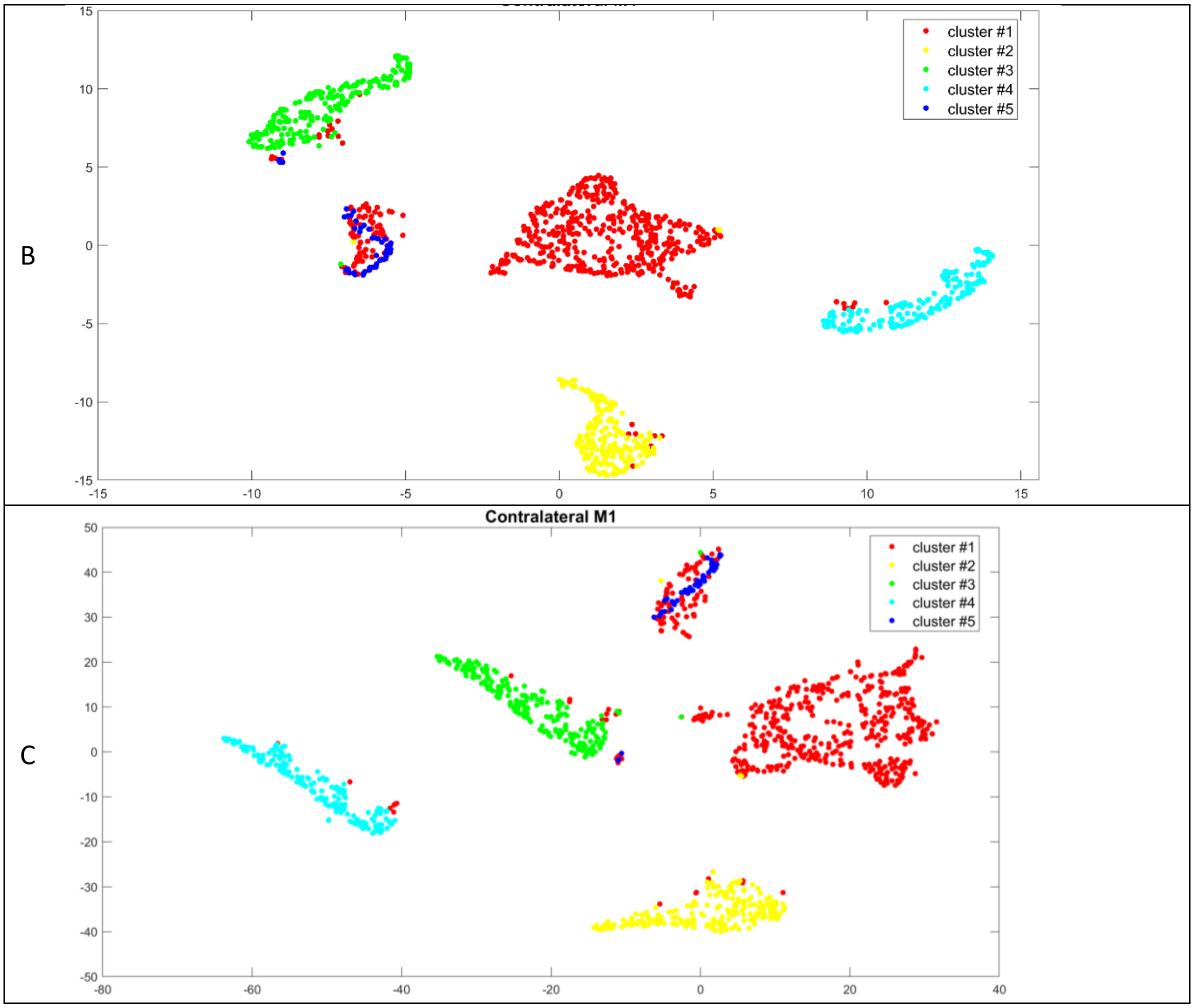

Figure S4: Robust clusters for contralateral Panel A using principal component analysis (A), UMAP (B) and t-SNE (C)

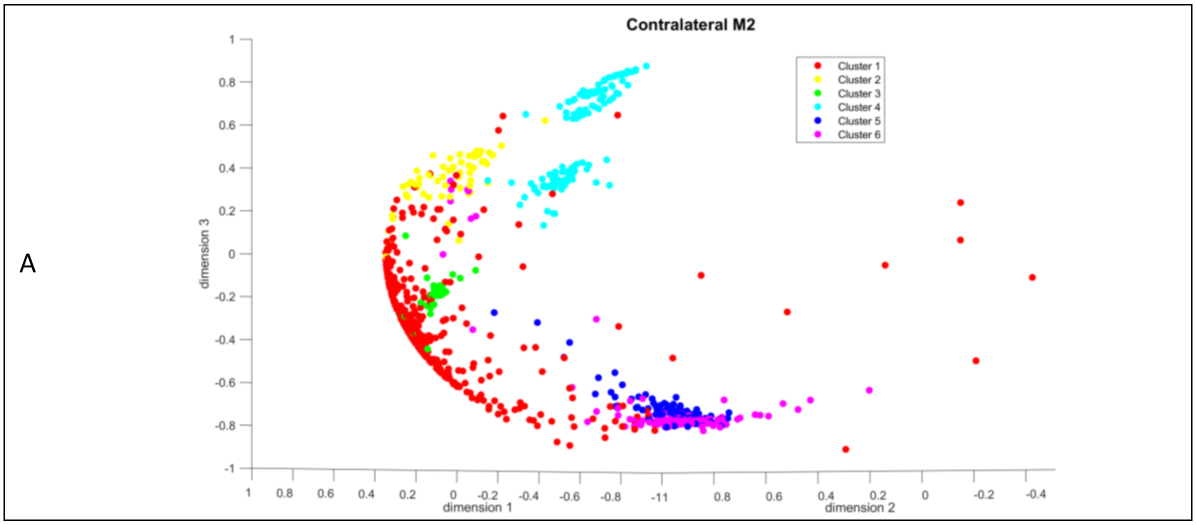

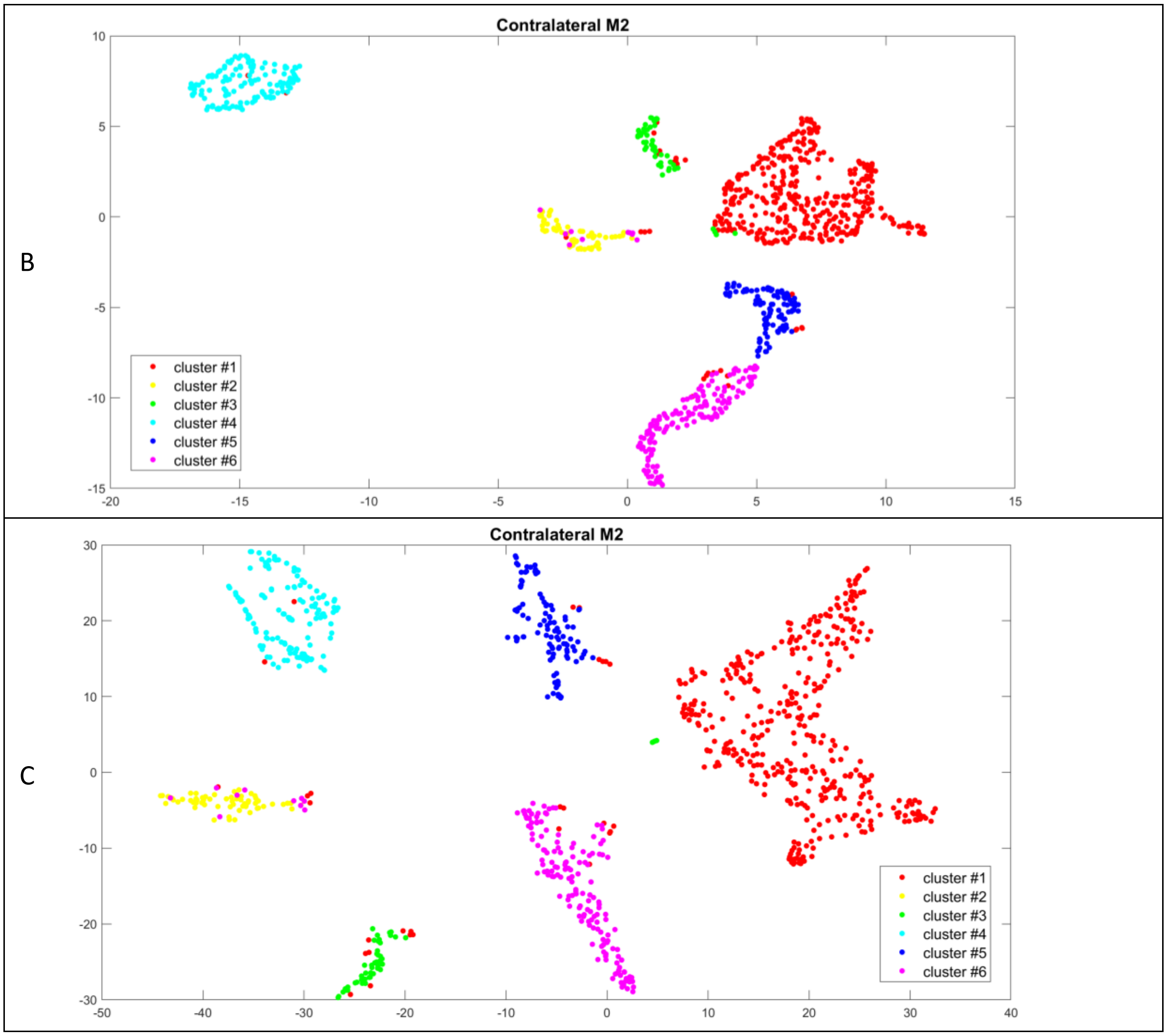

Figure S5: Robust clusters for contralateral Panel B using principal component analysis (A), UMAP (B) and t-SNE (C)

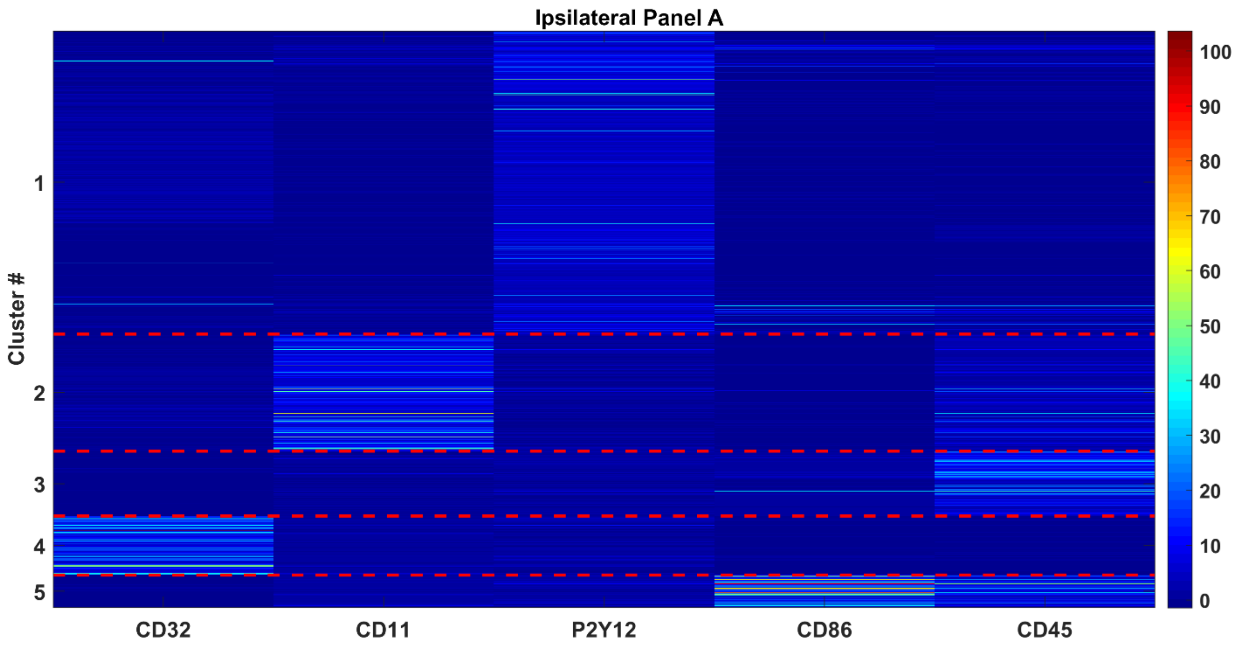

Figure S6: Cluster characteristics for ipsilateral Panel A across the different markers. Scale is in standard deviations from sham.

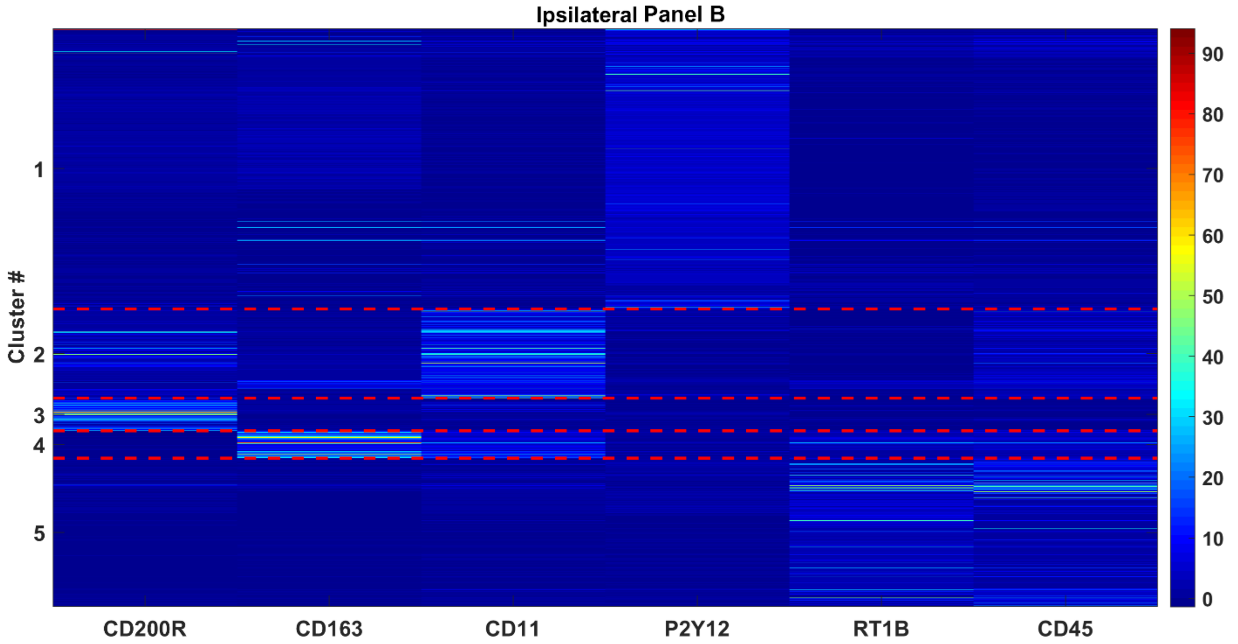

Figure S7: Cluster characteristics for ipsilateral Panel B across the different markers. Scale is in standard deviations from sham.

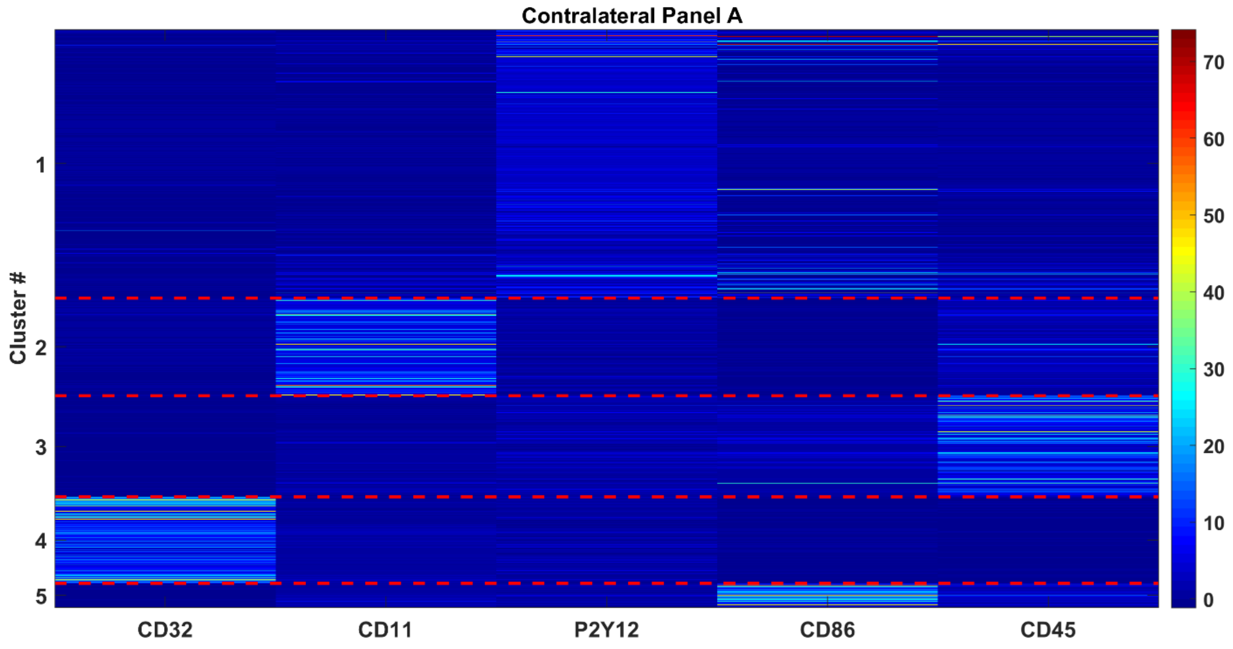

Figure S8: Cluster characteristics for contralateral Panel A across the different markers. Scale is in standard deviations from sham.

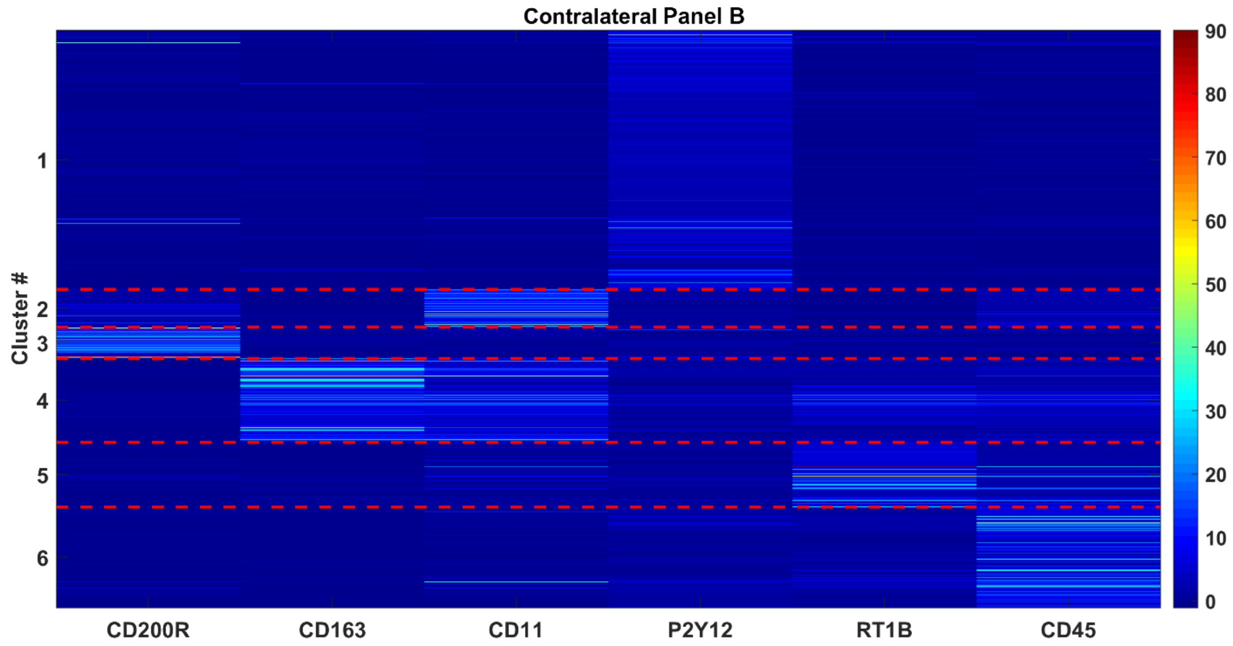

Figure S9: Cluster characteristics for contralateral Panel B across the different markers. Scale is in standard deviations from sham.

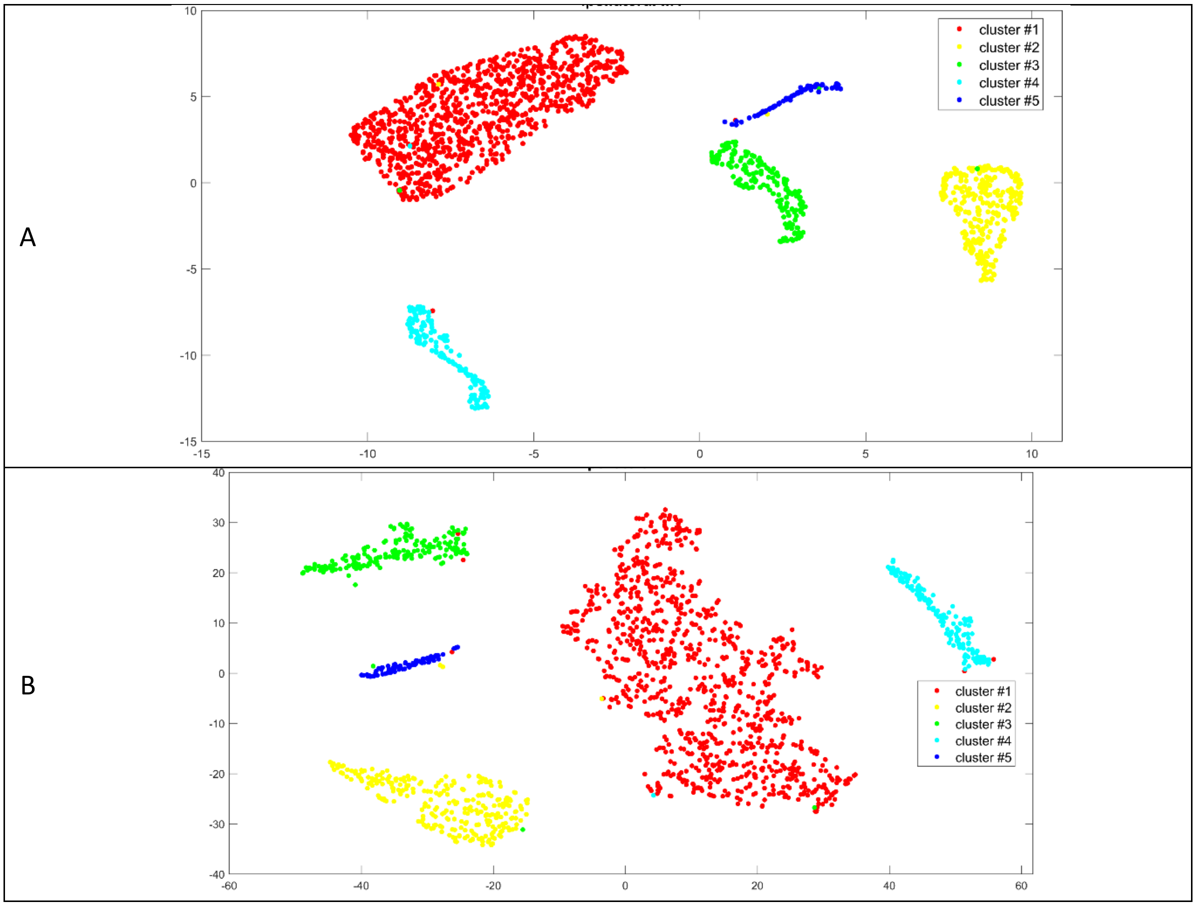

Figure S10: Expanded clusters for ipsilateral Panel A using UMAP (A) and t-SNE (B). For viewing purposes, a random sample of 1000 of cluster #1 cells are displayed.

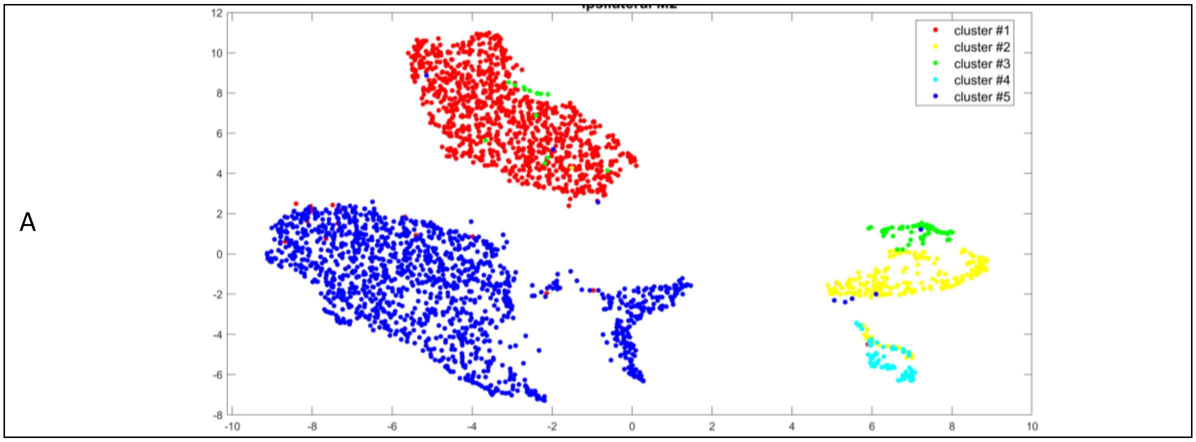

B

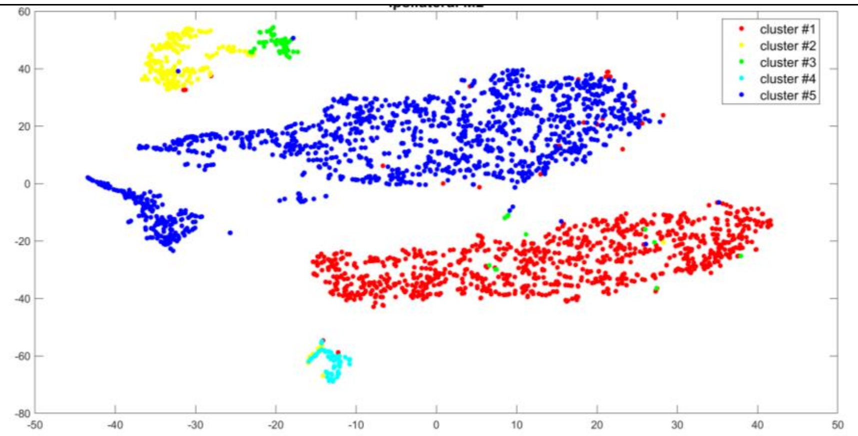

Figure S11: Expanded clusters for ipsilateral Panel B using UMAP (A) and t-SNE (B). For viewing purposes, a random sample of 1000 of cluster #1 cells are displayed.

A

B

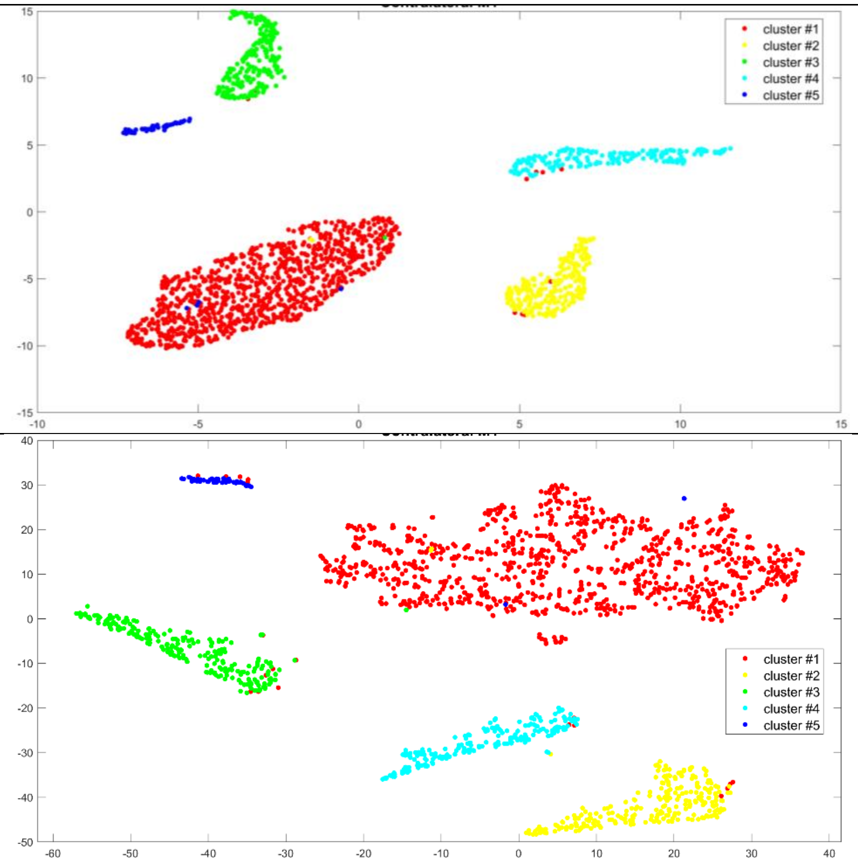

Figure S12: Expanded clusters for contralateral Panel A using UMAP (A) and t-SNE (B). For viewing purposes, a random sample of 1000 of cluster #1 cells are displayed.

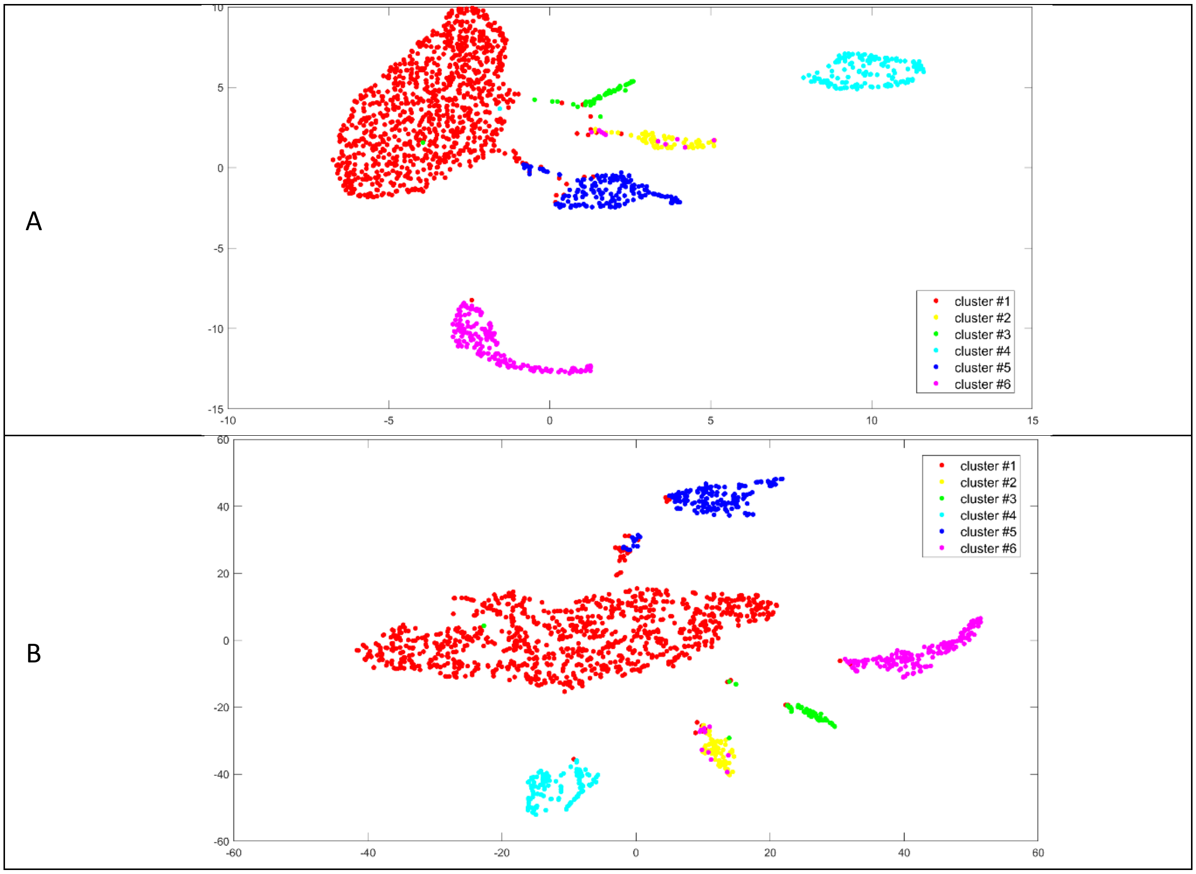

Figure S13: Expanded clusters for contralateral Panel B using UMAP (A) and t-SNE (B). For viewing purposes, a random sample of 1000 of cluster #1 cells are displayed.
